# Supplementary material for: Resolving the full spectrum of human genome variation using Linked-Reads
Source: Genome Res. 2019 Apr;29(4):635–45. doi: 10.1101/gr.234443.118 (PMC6442396; doi:10.1101/gr.234443.118)
Supplement: Supplemental Material [file supp_gr.234443.118_Supplemental_longranger-2.2.zip › longranger-2.2.tar.bz2!/lr2.2-public/lib/stan/cmdstan/stan/doc/api/html/agrad_2fwd_2functions_2rising__factorial_8hpp_source.html]

Stan: src/stan/agrad/fwd/functions/rising\_factorial.hpp Source File


|  |  |
| --- | --- |
|  | Stan  2.5.0  probability, sampling & optimization |


- Main Page
- Related Pages
- Namespaces
- Classes
- Files

- File List
- File Members

All Classes Namespaces Files Functions Variables Typedefs Enumerations Enumerator Friends Macros Pages

- src
- stan
- agrad
- fwd
- functions

rising\_factorial.hpp

Go to the documentation of this file.

1 #ifndef STAN\_\_AGRAD\_\_FWD\_\_FUNCTIONS\_\_RISING\_FACTORIAL\_HPP

2 #define STAN\_\_AGRAD\_\_FWD\_\_FUNCTIONS\_\_RISING\_FACTORIAL\_HPP

3

4 #include <stan/agrad/fwd/fvar.hpp>

5 #include <stan/meta/traits.hpp>

6 #include <stan/math/functions/rising\_factorial.hpp>

7 #include <boost/math/special\_functions/digamma.hpp>

8

9 namespace stan {

10

11  namespace agrad {

12

13  template<typename T>

14  inline

15  fvar<T>

16  rising\_factorial(const fvar<T>& x, const fvar<T>& n) {

17  using stan::math::rising\_factorial;

18  using boost::math::digamma;

19

20  T rising\_fact(rising\_factorial(x.val\_,n.val\_));

21  return fvar<T>(rising\_fact, rising\_fact \* (digamma(x.val\_ + n.val\_)

22  \* (x.d\_ + n.d\_) - digamma(x.val\_) \* x.d\_));

23  }

24

25  template<typename T>

26  inline

27  fvar<T>

28  rising\_factorial(const fvar<T>& x, const double n) {

29  using stan::math::rising\_factorial;

30  using boost::math::digamma;

31

32  T rising\_fact(rising\_factorial(x.val\_,n));

33  return fvar<T>(rising\_fact,

34  rising\_fact \* x.d\_ \* (digamma(x.val\_ + n) - digamma(x.val\_)));

35  }

36

37  template<typename T>

38  inline

39  fvar<T>

40  rising\_factorial(const double x, const fvar<T>& n) {

41  using stan::math::rising\_factorial;

42  using boost::math::digamma;

43

44  T rising\_fact(rising\_factorial(x,n.val\_));

45  return fvar<T>(rising\_fact,

46  rising\_fact \* (digamma(x + n.val\_) \* n.d\_));

47  }

48  }

49 }

50 #endif

rising\_factorial.hpp

stan::agrad::fvar

**Definition:** fvar.hpp:13

traits.hpp

fvar.hpp

stan::math::rising\_factorial

boost::math::tools::promote\_args< T1, T2 >::type rising\_factorial(const T1 x, const T2 n)

**Definition:** rising\_factorial.hpp:53

stan::agrad::rising\_factorial

fvar< T > rising\_factorial(const fvar< T > &x, const fvar< T > &n)

**Definition:** rising\_factorial.hpp:16

stan::agrad::digamma

fvar< T > digamma(const fvar< T > &x)

**Definition:** digamma.hpp:16

stan::agrad::fvar::d\_

T d\_

**Definition:** fvar.hpp:16

stan::agrad::fvar::val\_

T val\_

**Definition:** fvar.hpp:15


---

[ Stan Home Page ]

*© 2011–2014,
Stan Development Team.*
